# Who should lead a trauma team: Surgeon or non-surgeon? A systematic review and meta-analysis

**DOI:** 10.5249/jivr.v9i2.874

**Published:** 2017-07

**Authors:** Shahab Hajibandeh, Shahin Hajibandeh

**Affiliations:** ^*a*^Department of General Surgery, North Manchester General Hospital, Manchester, UK.; ^*b*Department of General Surgery, Royal Albert Edward Infirmary, Wigan, UK. ^

**Keywords:** Wounds and injuries, Leadership, Emergency care

## Abstract

**Background:**

Presence of a trauma team leader (TTL) in the trauma team is associated with positive patient outcomes in major trauma. The TTL is traditionally a surgeon who coordinates the resuscitation and ensures adherence to Advanced Trauma Life Support (ATLS) guidelines. The necessity of routine surgical leadership in the resuscitative component of trauma care has been questioned by some authors. Therefore, it remains controversial who should lead the trauma team. We aimed to evaluate outcomes associated with surgeon versus non-surgeon TTLs in management of trauma patients.

**Methods:**

In accordance with Preferred Reporting Items for Systematic Reviews and Meta-Analyses (PRISMA) statement standards, we performed a systematic review. Electronic databases MEDLINE, EMBASE, CINAHL and the Cochrane Central Register of Controlled Trials (CENTRAL) were searched to identify randomized and non-randomized studies investigating outcomes associated with surgeon versus non-surgeon TTL in management of trauma patients. The Newcastle-Ottawa scale was used to assess the methodological quality and risk of bias of the selected studies. Fixed-effect model was applied to calculate pooled outcome data.

**Results:**

Three retrospective cohort studies, enrolling 2,519 adult major trauma patients, were included. Our analysis showed that there was no difference in survival [odds ratio (OR): 0.82, 95% confidence interval (CI) 0.61-1.10, P=0.19] and length of stay when trauma team was led by surgeon or non-surgeon TTLs; however, fewer injuries were missed when the trauma team was led by a surgeon (OR: 0.48, 95% CI 0.25-0.92, P=0.03).

**Conclusions:**

Despite constant debate, the comparative evidence about outcomes associated with surgeon and non-surgeon trauma team leader is insufficient. The best available evidence suggests that there is no significant difference in outcomes of surgeon or non-surgeon trauma team leaders. High quality randomized controlled trials are required to compare the effectiveness of surgeon and non-surgeon trauma team leaders in order to resolve the controversy about who should lead the trauma team. Clinically significant missed injuries should be considered as important outcome in future studies.

## Introduction

Trauma is a leading cause of death and disability worldwide.^1^ The introduction of trauma teams has improved outcomes of the initial assessment and resuscitation of trauma patients. ^2,3^ A trauma team is a multidisciplinary team consisting of a group of individuals from various specialties including anesthesia, emergency medicine, surgery, nursing and support staff. 

Presence of a trauma team leader (TTL) in the trauma team is associated with positive patient outcomes in major trauma.^2,4^ A TTL should be familiar with trauma triage, be aware of trauma care protocol, be exposed to evidence-based studies, be adept to kinematics of various injuries, be able to execute multiple tasks of trauma resuscitation, be capable to diagnose cases that need immediate surgical interventions, and be skilled in various routine and critical care issues. A TTL should not only provide complete, coordinated and efficient care but also enhance the entire trauma system through a variety of activities, including education, secondary injury prevention and control, and injury surveillance.^5^

The TTL is traditionally a surgeon who coordinates the resuscitation and ensures adherence to Advanced Trauma Life Support (ATLS) guidelines.^6^ Considering the ongoing evolution of care in trauma management and the training of nonsurgical specialties in trauma care, the necessity of routine surgical leadership in the resuscitative component of trauma care has been questioned by some authors due to lack of objective evidence in favor of mandatory surgical leadership of trauma teams.^5,7-9^ Therefore, it remains controversial who should lead the trauma team.

Our objective was to perform a systematic review of the literature and conduct a meta-analysis of outcomes associated with surgeon versus non-surgeon TTLs in management of trauma patients. We considered trauma patients as participants of interest; surgeon TTL as intervention of interest; non-surgeon TTL as comparison of interest; survival, missed injuries, and length of stay as outcomes of interest; and randomized controlled trials (RCTs) and observational studies as study designs of interest. The robustness and quality of the available evidence was evaluated in a systematic and explicit approach with consideration of consistency and generalizability of the results.

## Methods

This systematic review was performed according to an agreed predefined protocol. The review was conducted and presented according to Preferred Reporting Items for Systematic Reviews and Meta-Analyses (PRISMA) statement standards.^10^

**Eligibility criteria**

We planned to include all randomized controlled trials and observational studies investigating outcomes associated with surgeon versus non-surgeon TTLs in management of trauma patients. A surgeon TTL was considered as intervention of interest and a non-surgeon TTL was considered as comparator.

**Outcome measures**

Survival at discharge was considered as primary outcome measure. The secondary outcome measures included length of stay, and missed injury rate. Missed injury was defined as an injury not detected by primary, secondary, and tertiary surveys in the initial 24 hours after presentation.

**Literature search strategy**

Two authors (Shahab H, Shahin H) independently searched the following electronic databases: MEDLINE, EMBASE, CINAHL and the Cochrane Central Register of Controlled Trials (CENTRAL). The last search was run on 10 June 2016. The details of the search strategy, which was adapted according to thesaurus headings, search operators and limits in each of the above databases, are appended in Appendix 1. In addition, the following trial databases were searched for details of ongoing and unpublished studies: World Health Organization International Clinical Trials Registry http://apps.who.int/trialsearch/, ClinicalTrials.gov http://clinicaltrials.gov/, ISRCTN Register http://www.isrctn.com/. We searched the bibliographic lists of relevant articles and reviews for further potentially eligible trials. No language restrictions were applied in our search strategies.

**Appendix 1:** Appendix I

Search No	Search strategy†
#1	MeSH descriptor: [wounds and injuries] explode all trees
#2	wound* or trauma* or injur* or fracture* or burn* or stab* or shot* or shoot* or lacerat* or accident*): TI,AB,KW
#3	miss* injur*: TI,AB,KW
#4	#1 OR #2 OR #3
#5	MeSH descriptor: [leadership] explode all trees
#6	leader: TI,AB,KW
#7	“trauma team leader”: TI,AB,KW
#8	TTL: TI,AB,KW
#9	#5 OR #6 OR #7 OR #8
#10	#4 AND #9

† This search strategy was adopted for following databases: MEDLINE, EMBASE, CINAHL and the Cochrane Central Register of Controlled Trials (CENTRAL)

**Study selection**

Two authors (SH, SH) independently assessed the title and abstract of articles identified from the literature searches. The full-texts of relevant reports were retrieved and those articles that met the eligibility criteria of our review were selected. We resolved any discrepancies in study selection by discussion between the authors. An independent third reviewer was consulted in the event of disagreement.

**Data collection**

We created an electronic data extraction spreadsheet in line with the Cochrane's data collection form for intervention reviews. We pilot-tested the spreadsheet in randomly selected articles and adjusted it accordingly. Our data extraction spreadsheet included:

• Study-related data (first author, year of publication, country of origin of the corresponding author, journal in which the study was published, study design, study size, clinical condition of the study participants, number of trauma centers, and level of the trauma center)

• Baseline demographic and clinical information of the study populations (age, gender, and injury severity score)

• Primary and secondary outcome data

Two authors (Shahab H, Shahin H) independently collected and recorded data and resolved disagreements by discussion. If no agreement could be reached, a third reviewer was consulted.

**Methodological quality and risk of bias assessment**

The methodological quality and risk of bias of the included articles were assessed independently by two authors (Shahab H, Shahin H). We planned to use the Cochrane's tool^11^ and the Newcastle-Ottawa scale (NOS)^12^ for assessing the risk of bias of randomized trials and observational studies, respectively. The Cochrane’s tool assesses domains including selection bias, performance bias, detection bias, attrition bias, reporting bias, and other sources of bias and, for each individual domain, classifies studies into low, unclear, and high risk of bias. The NOS uses a star system with a maximum of nine stars to evaluate a study in three domains (8 items): the selection of the study groups, the comparability of the groups, and the ascertainment of outcome of interest. For each item of the scale, we judged each study as low risk (one star awarded) or high risk (no star awarded). We determined studies that received a score of nine stars to be of low risk of bias, studies that scored seven or eight stars to be of moderate risk, and those that scored six or less to be of high risk of bias. Disagreements were resolved by discussion between the two reviewers. If no agreement could be reached, a third reviewer acted as an adjudicator. A risk of bias graph was constructed to present the results.

**Data synthesis and statistical analyses**

For dichotomous outcome variables (survival, and missed injury rate), we calculated the odds ratio (OR) as the summary measure. The OR is the odds of an event in the surgeon TTL group compared to the non-surgeon TTL group. For survival, an OR of more than one would favor the surgeon TTL. For missed injury rate, an OR of less than one would favor the surgeon TTL. For continuous parameters (length of stay), we planned to calculate the mean difference (MD) between the two groups.

We used the individual patient as the unit of analysis. Information about dropouts, withdrawals and other missing data were recorded and, if not reported, we contacted the study authors where possible. The final analysis was based on intention-to-treat data from the individual clinical studies.

The Review Manager 5.3 software was used for data synthesis.^11^ Extracted data were entered into Review Manager by the first independent author (Shahab H) and checked by the second independent author (Shahin H). We used fixed effect modeling for analysis. We planned to apply random effects models if considerable heterogeneity among the studies, as defined by Higgins et al,^11^ was identified. The results were reported in a forest plot with 95% confidence intervals (CIs).

Heterogeneity among the studies was assessed using the Cochran Q test (χ2). We quantified inconsistency by calculating I2 and interpreted it using the following guide: 0% to 25% may represent low heterogeneity; 25% to 75%: may represent moderate heterogeneity; and 75% to 100% may represent considerable heterogeneity. We planned to calculate the Egger’s regression intercept to formally assess reporting bias using the Comprehensive Meta-Analysis (CMA) software (Biostat, Englewood, NJ) , as long as a sufficient number of studies (more than 3) were available. Also, we planned to construct funnel plots and evaluate their symmetry to visually assess publication bias, as long as a sufficient number of studies (more than 10) were available.

**Sensitivity analyses**

We planned to perform additional analyses to explore potential sources of heterogeneity and assess the robustness of our results. For each outcome, we planned to repeat the primary analysis using random effects models and fixed effect models. In addition, we calculated the pooled risk ratio (RR) and risk difference (RD) for each dichotomous variable. We assessed the effect of each study on the overall effect size and heterogeneity by repeating the analysis after removing one study at a time. Also, we planned to perform separate analyses for studies with low, moderate, or high risk of bias to assess the change in direction of the effect size.

## Results

**Literature search results**

**Figure 1:**
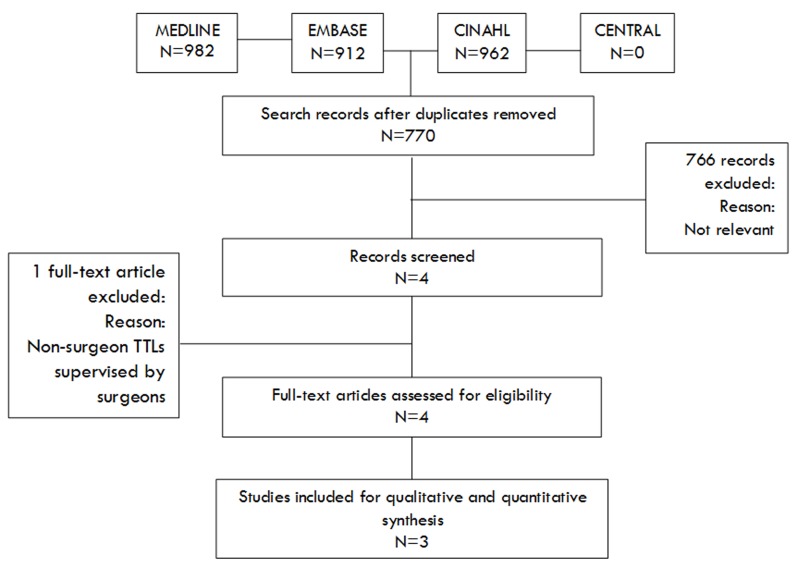
Study flow diagram.

Searches of electronic databases identified 770 articles of which three studies^13-15^ were eligible for this review. These included three retrospective cohort studies,^13-15^ enrolling a total of 2,519 adult major trauma patients. One study was excluded because no-surgeon TTL acted under supervision by surgeons. ^16^ The literature search flow chart and baseline characteristics of the included studies and population are demonstrated in Figure 1  and Table 1, respectively.

**Table 1:** Characteristics of the included studies and population

	Cummings 2007	Ahmed 2007	
Journal	CJEM	Ann Emerg Med	J Trauma Acute Care Surg
Country	Canada	Canada	Canada
Study design	Retrospective cohort	Retrospective cohort	Retrospective cohort
Population	Adult major blunt trauma patients	Mainly major blunt trauma patients	Mainly major blunt trauma patients
No of centers	2	1	1
Level I trauma center	No	Yes	Yes
Sample size	1412	807	300
Age, years	Surgeon Group: 43 Non-surgeon Group:45(median)	Surgeon Group: 34 Non-surgeon Group:36 (median)	Surgeon Group: 41 Non-surgeon Group:40 (median)
Male gender, %	Surgeon Group: 75.6% Non-surgeon Group: 74,4%	Surgeon Group: 75.7% Non-surgeon Group:77.1%	Surgeon Group: 72.2% Non-surgeon Group:73.0%
ISS	Surgeon Group: 23.7 Non-surgeon Group:23.1(mean)	Surgeon Group: 23 Non-surgeon Group:21 (median)	Surgeon Group: 22 Non-surgeon Group:25 (median)

ISS: Injury severity score

**Description of included studies **

Cummings 2007^13^ was a retrospective cohort study that enrolled 1,412 trauma patients in two major trauma centers. This study included all adult trauma patients who had an Injury Severity Score (ISS) ≥ 12 and a recorded Revised Trauma Score (RTS). Patients who had penetrating trauma or had missing information on age, sex, ISS, RTS and TTL-type were excluded from this study. The surgeon TTLs at both centers were Royal College fellowship certified general surgeons, with variable amounts of additional postgraduate specialty training in trauma care. The non-surgeon TTLs at both centers were emergency physicians who were either Royal College fellowship certified physicians (FRCP), or family physicians with 1 year of emergency training (CCFP-EM).^13^

Ahmed 2007^14^ was a retrospective cohort study that enrolled 807 trauma patients in a level I trauma center. This study included all adult trauma patients with a major blunt injury (ISS ≥12) or a major penetrating injury (ISS ≥9). Patients with major burns or with missing information about TTL-type were excluded from this study. The surgeon TTLs were either general surgeons, orthopedic surgeons, plastic surgeons, or thoracic surgeons. The non-surgeon TTLs were either emergency physicians or anaesthesiologists.^14^

Leeper 2013^15^ was a retrospective cohort study that enrolled 300 trauma patients in a level I trauma center. This study included all adult trauma patients who had an ISS > 12. Surgeon TTLs were principally general surgeons with subspecialty training in trauma, vascular, or thoracic surgery. The non-surgeon TTLs were either emergency physicians or critical care physicians.^15^

**Risk of bias in included studies**

**Figure 2:**
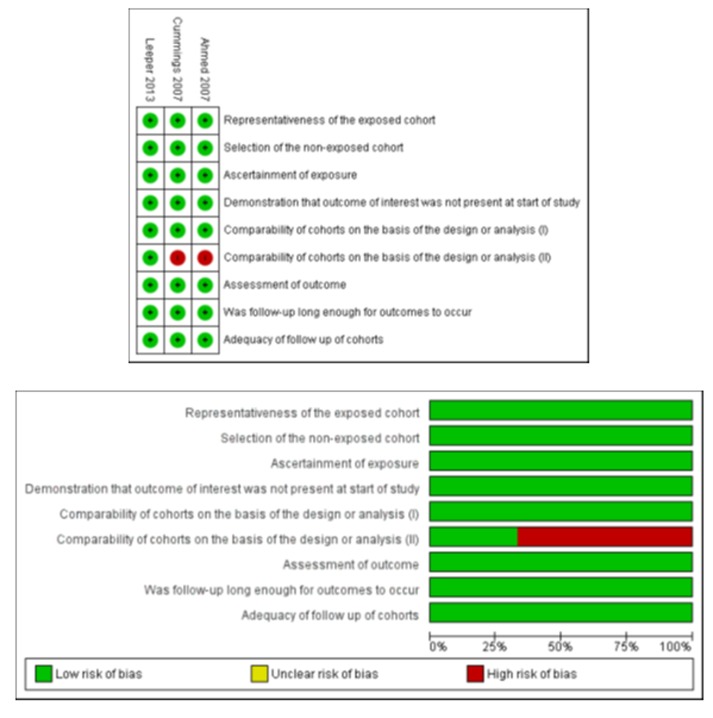
Risk of bias summary and graph showing authors’ judgments about each Newcastle-Ottawa scale item for each included study

One study^15^ was judged to be of low risk of bias and two studies^13,14^ were judged to be of moderate risk of bias. The summary and results of methodological quality assessment are demonstrated graphically in Figure 2.

**Outcome synthesis**

**Figure 3:**
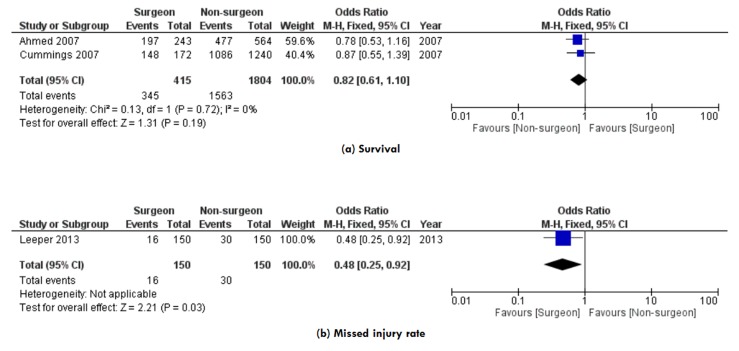
Forest plots of comparison of a) Survival, and (b) Missed injury rate. The solid squares denote the odds ratios (ORs), the horizontal lines represent the 95% confidence intervals (CIs), and the diamond denotes the pooled OR. M-H, Mantel Haenszel test.

**Survival. **Survival was reported in two studies,^13,14^ enrolling 2,219 patients (Figure 3a). There was no significant difference in survival between the surgeon TTL and non-surgeon TTL groups (OR: 0.82, 95% CI 0.61-1.10, P=0.19). A low level of heterogeneity among the studies existed (I2=0%, P=0.72).

**Length of stay. ** Length of stay was reported in two studies, ^13,14^ enrolling 2,219 patients. We did not form a meta-analytical model for this outcome because of heterogeneous outcome definitions (Cummings 2007^13^ reported the length of stay in the Emergency Department whereas Ahmed 2007^14^ reported the length of stay in the hospital). There was no significant difference in the median Emergency Department length of stay between surgeon TTL and non-surgeon TTL groups in Cummings 2007^13^ (4.5 hours versus 5.5 hours). Consistent with this, Ahmed 2007^14^ found to significant difference in the median hospital length of stay between surgeon TTL and non-surgeon TTL groups (12 days versus 12 days).

**Missed injury rate. **Missed injury rate was reported in one study,^15^ enrolling 300 patients (Figure 3b). Fewer injuries were missed when the trauma team was led by a surgeon compared to when the trauma team was led by a non-surgeon leader (OR: 0.48, 95% CI 0.25-0.92, P=0.03). The heterogeneity assessment was not applicable for this outcome as we analyzed data from one study.

**Sensitivity analysis**

Considering the limited number of included studies, we performed sensitivity analyses only for one outcome (survival) (Table 2). The use of random-effects or fixed-effect models did not affect the direction of the effect size for survival. Moreover, the direction of effect size remained unchanged when RRs or RDs were calculated. Removal of one study at a time did not affect the overall heterogeneity and the direction of the effect size.

**Table 2:** Results of sensitivity analysis for survival

Description of analysis	Number of studies	Number of patients	OR (95% CI)	P value	I2
Cummings 2017 removed	1	807	0.78 [0.53, 1.16]	0.22	Not applicable
Ahmed 2007 removed	1	1412	0.87 [0.55, 1.39]	0.007	Not applicable
Random-effects model	2	2219	0.82 [0.61, 1.11]	0.19	0%
Calculating risk ratio instead of OR	2	2219	0.97 [0.92, 1.02]*	0.21	0%
Calculating risk difference instead of OR	2	2219	-0.03 [-0.07, 0.01]†	0.21	0%

OR: odds ratio; CI: confidence interval *Risk ratio calculated instead of OR †Risk difference calculated instead of OR

## Discussion

We conducted a systematic review of the literature and meta-analysis of reported outcomes associated with surgeon versus non-surgeon trauma team leaders in management of trauma patients. We included three retrospective cohort studies,^13-15^ enrolling a total of 2,519 adult major trauma patients. Our analysis showed that there was no difference in survival and length of stay when trauma team was led by surgeon or non-surgeon TTLs; however, fewer injuries were missed when the trauma team was led by a surgeon. The included population was homogenous in terms of baseline demographics. A low level of between-study heterogeneity was identified. We used the fixed-effect model for analysis of the outcomes. The overall heterogeneity and direction of effect size remained consistent throughout our sensitivity analyses. Nevertheless, due to a limited number of available studies, the currently available comparative evidence is not adequately robust to reach definite conclusions.

There was no difference in survival when the trauma team was lead by a surgeon or no-surgeon. The number of patients was significantly greater in the non-surgeon TTL group than surgeon TTL group. Considering that a large number of trauma patients would survive following the initial treatment,^17^ the smaller number of patients in surgeon TTL group might have led to underestimation of survival rate in this group. The similar baseline demographics, ISS, and type of injury of the included patients may explain the low between-study heterogeneity for survival in our study.

Although the best available evidence supports the safety of non-surgeon TTL in terms of survival, the available evidence suggests that leading the trauma team by non-surgeon TTLs might be associated with increased risk of missed injuries. Missed injuries are considered as an important issue in trauma patients and can lead to significant morbidity and even mortality.^18^ They are evidently present after primary and secondary surveys. ^19^ The results from study of Leeper et al. ^15^ suggests that presence of a non-surgeon TTL is an independent predictor of missed injuries. This may be explained by the fact that surgeon TTLs are involved in the inpatient and follow-up care of trauma patients, and are likely to have better awareness regarding the detection of missed injuries and the morbidity associated with such injuries whereas non-surgeon TTLs’ awareness regarding missed injuries (detected later in patient’s care) is mainly limited to morbidity and mortality meetings. In Leeper 2013, ^15^ non-surgeon TTLs ordered more computed tomography (CT) imaging compared to surgeon TTLs; therefore, higher missed injury rate was unlikely to be affected by the intensity of the initial diagnostic workup in this study. Nevertheless, the above finding is on the basis of a single retrospective observational study which is subject to selection bias therefore, high quality evidence is required to reach definite conclusions. Moreover, missed injuries may be minor and self-limiting injuries that only require conservative management. Therefore, clinically significant missed injuries should be distinguished from minor injuries.

The American College of Surgeons recommends that trauma team leader should be a surgeon.^20^ However, the presence of a surgeon at trauma resuscitations has been considered superfluous by some authors arguing that non-surgeon TTLs are able to effectively manage trauma patient resuscitative care.^21-23^ Although the best available evidence shows that survival rate and length of stay associated with non-surgeon TTLs are comparable to those associated with surgeon TTLs, this may not represent overall effectiveness of non-surgeon physicians as leaders or providers of trauma care. Clinically significant missed injury as a crucial outcome measure for monitoring the TTL performance has been inadequately investigated by authors in the current literature; therefore, further evidence is required before any policy changes can be considered.

In this study, we used a systematic and explicit approach with consideration of consistency and generalizability of the results to provide a summary of the best available evidence and assess the risk of bias of relevant studies. We aimed to investigate implications for clinical practice and identify areas for future research. The reported outcomes of our review and analysis should be viewed and interpreted in the context of inherent limitations. None of the included studies were RCT which is the gold standard study design for the purpose of our study. This will affect the statistical robustness of our meta-analysis. The best available evidence is from retrospective cohort studies that are inevitably subject to selection bias. There were a limited number of eligible studies for this review; therefore, the available evidence was insufficient to draw solid conclusions on the comparative efficacy of surgeon versus non-surgeon TTLs. All of the included studies were conducted in Canada. Considering that training of surgeon or non-surgeon TTLs vary considerably in different countries, this may affect the generalizability of our findings. Moreover, training of anesthesiologists, emergency medicine physicians, and critical care physicians are different; therefore, the performance of each specialty as TTL may be different. The available data did not allow for subgroup analysis based on different non-surgical specialties within non-surgeon TLL group. This can potentially subject our results to bias. Also, the available data did not allow us to perform subgroup analysis based on parameters such as ISS. Finally, as discussed earlier, the smaller number of patients in the surgeon TTL group might have led to underestimation of survival rate in this group.

## Conclusion

Despite constant debate, the comparative evidence about outcomes associated with surgeon and non-surgeon trauma team leader is insufficient. The best available evidence suggests that there is no significant difference in outcomes of surgeon or non-surgeon trauma team leaders. High quality randomized controlled trials are required to compare the effectiveness of surgeon and non-surgeon trauma team leaders in order to resolve the controversy about who should lead the trauma team. Clinically significant missed injuries should be considered as important outcome in future studies. 
